# Prognostic Value of MACC1 in Digestive System Neoplasms: A Systematic Review and Meta-Analysis

**DOI:** 10.1155/2015/252043

**Published:** 2015-05-19

**Authors:** Zhenzhen Wu, Rui Zhou, Yuqi Su, Li Sun, Yulin Liao, Wangjun Liao

**Affiliations:** ^1^Department of Oncology, Nanfang Hospital, Southern Medical University, Guangzhou 510515, China; ^2^Department of Cardiology, Nanfang Hospital, Southern Medical University, Guangzhou 510515, China

## Abstract

Metastasis associated in colon cancer 1 (MACC1), a newly identified oncogene, has been associated with poor survival of cancer patients by multiple studies. However, the prognostic value of MACC1 in digestive system neoplasms needs systematic evidence to verify. Therefore, we aimed to provide further evidence on this topic by systematic review and meta-analysis. Literature search was conducted in multiple databases and eligible studies analyzing survival data and MACC1 expression were included for meta-analysis. Hazard ratio (HR) for clinical outcome was chosen as an effect measure of interest. According to our inclusion criteria, 18 studies with a total of 2,948 patients were identified. Pooled HRs indicated that high MACC1 expression significantly correlates with poorer OS in patients with digestive system neoplasms (HR = 1.94; 95% CI: 1.49–2.53) as well as poorer relapse-free survival (HR = 1.94, 95% CI: 1.33–2.82). The results of subgroup studies categorized by methodology, anatomic structure, and cancer subtype for pooled OS were all consistent with the overall pooled HR for OS as well. No publication bias was detected according to test of funnel plot asymmetry and Egger's test. In conclusion, high MACC1 expression may serve as a prognostic biomarker to guide individualized management in clinical practice for digestive system neoplasms.

## 1. Introduction

Digestive system neoplasms, including colorectal cancer (CRC), gastric cancer (GC), esophageal cancer (EC), pancreatic cancer (PC), and hepatocellular carcinoma (HCC), are among the top ten diseases for worldwide morbidity and mortality rate [1]. To precisely predict the prognosis and therapeutic effects of patients with digestive system neoplasms, efficient and approachable biomarkers are needed in clinical practice. Although numerous biomarkers involved in digestive system neoplasms have been identified, only a few have been well validated for clinical usage [2]. Therefore, efforts to develop new reliable prognostic markers should be made to help modify clinical management for patients with digestive system neoplasms.

Metastasis associated in colon cancer 1 (MACC1) was newly identified as an oncogene regulating the hepatocyte growth factor/met tyrosine kinase receptor epidermal growth factor (HGF/c-Met) pathway which is well recognized to promote carcinogenesis and tumor progression by facilitating migration and invasion as well as suppressing apoptosis of cancer cells [3]. Studies confirmed that MACC1 plays tumor-promoting role [3, 4], indicating that it might be a potential risk factor for adverse clinical outcome. MACC1 overexpression has been reported to promote cell proliferation, HGF-triggered cell scattering, and cell migration and invasion in both cell cultures and xenograft models [3]. In contrast, silencing MACC1 can attenuate tumor cell growth and metastasis [5, 6]. Moreover, metabolic stress in GC can upregulate MACC1 expression and the overexpression of MACC1 sustains GC cell growth against metabolic stress by facilitating the Warburg effect [7]. In addition, we recently showed that MACC1 upregulated VEGF-C/VEGF-D secretion to promote lymphangiogenesis via c-Met signaling [8]. Regarding clinical significance, MACC1 was first reported to be an independent prognostic indicator of metastasis formation and metastasis-free survival in colon cancer [3]. Currently, MACC1 expression has been further proven to contribute to unfavorable clinical outcome of patients with gastric cancer, esophageal cancer, and hepatocellular carcinoma [5, 9, 10]. In addition, a recent meta-analysis that included a total of 20 eligible studies with patients showed that overexpression of MACC1 was significantly associated with poorer survival in multiple solid tumors, including lung cancer, breast cancer, and glioma [11].

Although multiple studies demonstrate that MACC1 overexpression is correlated with worse clinical outcomes in cancer patients, no systematic evidence has been provided to verify the prognostic value of MACC1 in digestive system neoplasms. Here we synthesize the existing literature to evaluate the prognostic value of MACC1 in digestive system neoplasms.

## 2. Materials and Methods

### 2.1. Data Source and Literature Search

We performed systematic literature search through PubMed, EMBASE, Web of Science, and Chinese BioMedical Literature Database (CBM) and extracted all published articles related to MACC1 expression in digestive system neoplasms by January 29, 2015. The search strategy was composed using the following keywords in various forms and combinations in order to yield high sensitivity: “MACC1,” “metastasis-associated with colon cancer 1,” “cancer,” “carcinoma,” “tumor,” “neoplasm,” and “malignancy.” Both MeSH terms and free text words for synonyms were applied during the search. Additional manual searching was conducted for supplementation on this topic as well. Also the search was supplemented by consulting current contents, reviews, textbooks, specialized registers, or experts in the particular field of study and by reviewing the references. To obtain as many records as possible, no language restriction was set.

### 2.2. Study Selection

All the records retrieved were reviewed by two independent reviewers. Disagreement was resolved by discussion between the two reviewers or consultation with a third reviewer. Firstly, irrelevant records, reviews, case reports, studies on animals or cells, and studies of cancers not within the digestive system were excluded. Then abstracts of all remaining records were screened. Finally, eligible studies meeting the following criteria were included for full-text investigation: (1) proven diagnosis of digestive system neoplasms including CRC, GC, EC, PC, and HCC; (2) MACC1 expression evaluation using multiple methods based on serum or tissue specimens; (3) reported survival data stratified by MACC1 expression, including overall survival (OS), metastasis-free survival (MFS), relapse-free survival (RFS), and disease-free survival (DFS); (4) the most recent, largest, or most complete study for duplicate population. To minimize risk of bias, no language or time limitation was set.

### 2.3. Data Extraction

Two reviewers (Zhenzhen Wu and Rui Zhou) independently extracted relevant details from the included studies. Disagreement was settled by reviewing the original article together with a third reviewer (Yuqi Su). The data elements contain but were not confined to the following: (1) general information, including title, author, source, country, language, year of publication, study design, and follow-up; (2) patient information consisting of inclusion criteria, sample size, age, and sex; (3) tumor data of cancer subtype and TNM staging; (4) method to determine MACC expression and number of patients stratified by MACC expression; (5) clinical outcome (OS, MFS, RFS, DFS, etc.) and its corresponding hazard ratios (HRs) with 95% confidential intervals (CI).

When HR was not directly reported, survival data were extracted from amplified K-M curves by an open digitizing program (Engauge Digitizer) that converts curves into numbers at specific time intervals. The estimated HR and corresponding 95% CI were calculated via free available calculations spreadsheet by inputting the data extracted from K-M curves and estimating censoring using the minimum and maximum follow-up. If the number of observed deaths or disease progressions was available instead of the K-M curves, mathematical HR was estimated using established methods [27]. All studies not eligible for survival data extraction were excluded for meta-analysis.

### 2.4. Quality Assessment

Two independent reviewers (Zhenzhen Wu and Rui Zhou) assessed the quality of each study included for meta-analysis using the Newcastle-Ottawa Quality Assessment Scale (NOS) for non-RCT study [28]. The scale includes eight items which allows for assessment of patient selection, study comparability, and outcome of interest (see Table S2 in Supplementary Material available online at http://dx.doi.org/10.1155/2015/252043). Stars were given to high-quality elements and the total number of stars for each study was used to quantitate the overall study quality. Disagreement was resolved by consulting a third reviewer. A study with five or more stars was considered as a high-quality study [29].

### 2.5. Data Synthesis

The primary outcome set for this meta-analysis was OS associated with MACC1 expression in patients with digestive system neoplasms. Additionally, MFS, RFS, and DFS were adopted as secondary outcomes. HR with 95% CI was considered as the effect measure of interest. HR of greater than 1 was considered as an adverse outcome. Initially, the potential heterogeneity was demonstrated graphically in the Forest plot by comparing the overlapped parts of individual studies. Then Chi-squared (*χ* ^2^) test was performed to detect the heterogeneity. The *p* value and *I* ^2^ value were simultaneously calculated as the percentage of variability due to heterogeneity (*I* ^2^ < 25%: no heterogeneity; *I* ^2^ = 25–50%: moderate heterogeneity; *I* ^2^ > 50% or *p* > 0.01: large or extreme heterogeneity) [30]. For HR calculation, we applied a random-effect model for pooled dataset with large or extreme heterogeneity and a fixed-effect model for dataset with no or moderate heterogeneity.

### 2.6. Subgroup Analyses

We used subgroup analysis to assess whether the use of different subset led to different results. Accordingly, we classified studies into two subsets: MACC1 expression measurement (quantitative reverse transcriptase polymerase chain reaction (qRT-PCR) group and immunohistochemistry (IHC) group) and anatomic structure (gastrointestinal tract group and nongastrointestinal tract group). In addition, if there are more than two studies involved in the same cancer type, a subgroup analysis for this specific cancer type was also performed. The pooled HR estimates for each subgroup were calculated individually and were then compared to the overall HR estimate.

### 2.7. Sensitivity Analysis

To assess the stability of both overall pooled HR and subgroup analyses, sensitivity analysis was performed by sequentially removing individual study in each setting. Studies were removed one by one and the pooled HR was recalculated after the exclusion to identify the studies causing considerate fluctuation of HR estimate.

### 2.8. Assessment of Publication Bias

We firstly observed funnel plot asymmetry for publication bias. Then suspicious asymmetric distribution was subject to Egger's test and a *p* value less than 0.05 was considered as an indicator of significant publication bias [31]. Tests for funnel plot asymmetry and Egger's test should not be used when there are less than 10 studies included in the meta-analysis [31].

### 2.9. Statistical Analysis

All the statistical analyses were carried out by the RevMan software version 5.2 (The Cochrane Collaboration) and the META module of Stata version 12.0 (Stata Corporation, College Station, TX). *p* values for all comparisons were two-tailed and a *p* < 0.05 was considered as statistically significant for all tests except those for heterogeneity (*p* > 0.01). In metaregression analysis, a *p* < 0.05 indicates strong evidence for a certain factor contributing to the observed heterogeneity.

## 3. Results

### 3.1. Literature Search and Study Selection

**Figure 1:**
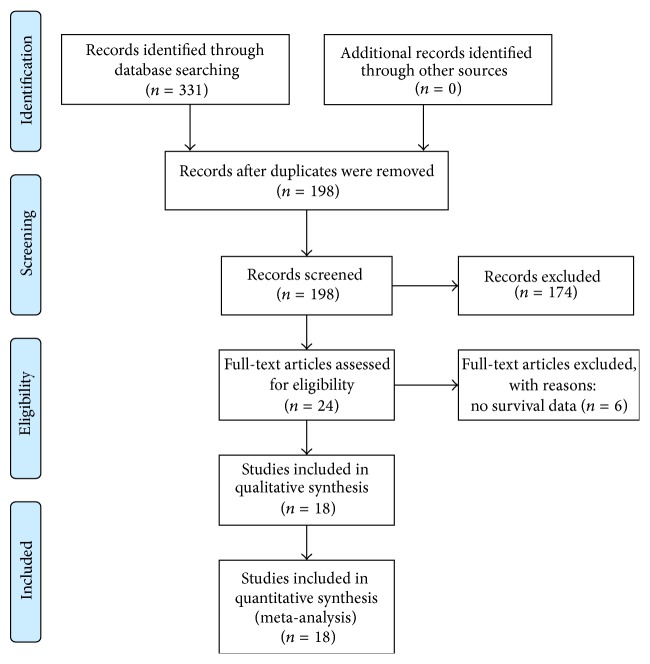
PRISMA flowchart demonstrating the selection process to identify eligible studies according to the PRISMA statement at http://www.prisma-statement.org/.

As illustrated in Figure 1, we identified 331 records in total through database searching, with 64 from PubMed, 103 from EMBASE, 107 from Web of Science, and 57 from Chinese BioMedical Literature Database (CBM) [32]. Manual searching yielded no additional record. After removing 133 duplicates, we initially screened the titles and abstracts of the 198 records left. 24 full-text articles were chosen for eligibility assessment in the next step. After thorough review with aforementioned inclusion and exclusion criteria, 6 studies [33–38] were excluded due to no MACC1 expression related survival data. The remaining 18 eligible studies were included in this meta-analysis. Among all eligible studies, 15 [5, 9, 10, 14–20, 22–24, 37] studies were subject to pooled OS; 2 [25, 26] studies were subject to pooled RFS, one of which [25] also reported DFS; and 1 study [3] reported MFS.

### 3.2. Study Characteristics and Quality Assessment

Table 1 lists the baseline characteristics of all 18 studies included in this meta-analysis. Patients were enrolled consecutively and followed up in 2 studies [3, 12] and all others studies were conducted retrospectively. The number of patients in each study ranges from 52 to 361, which renders data of total 2,948 patients available for meta-analysis. Included studies were first classified based on clinical outcome. 15 eligible studies were subject to pooled OS which was taken as our primary outcome set for this meta-analysis. Among the remaining 3 studies, 2 studies [25, 26] were subject to pooled RFS, one of which [25] also reported DFS, and 1 study [3] reported MFS. With respect to pooled OS, 15 studies consisted of 4 studies on CRC [14–16], 3 on GC [5, 17, 18], 1 on EC [9], and 7 on HCC [10, 19, 20, 22–24, 37]. The 2 studies for pooled RFS and 1 study reporting MFS were all performed in patients with CRC. No study on PC is available because the only study analyzing MACC1 expression in PC patients reports no OS data [36]. When considering TNM staging, 10 studies included patients with stage I to stage IV; 4 reported no tumor stage [19, 20, 24, 25]; 3 studies included patients with stage I to stage III [3, 9, 10]; and the remaining study [26] included patients with stage II to stage III. MACC1 expression measurement was not consistent in all studies. 8 studies [10, 12, 13, 19, 20, 24–26] applied qRT-PCR and the rest of studies chose IHC. Among the 8 studies measuring MACC1 via qRT-PCR, one used blood samples [12] and the rest tested tissue specimens. Additionally, the stratification of MACC1 expression varies among studies. Stein et al. [3, 12] used median value cut-off in their studies both in 2009 and in 2012. Qiu et al., Qu et al., Isella et al., and Kawamura et al. [10, 20, 25, 26] all used an arbitrary cut-off. Gao et al. [24] introduced ROC curve. In IHC measured studies, two different methods were used to determine the MACC1 cut-off values. 7 studies [5, 9, 14, 16, 18, 22, 23] derived a composite score by adding up both the staining intensity score and the integrals of the rate of positive cells, while 3 studies [15, 17, 21] referred to the proportion of positive immunoreactive cells. The proportion of “high” MACC1 expression ranged from 40.9% to 77.6% with a median value of 50%. For the 7 studies [3, 12, 14, 18, 21, 25, 26] without direct report of survival data, HRs with 95% CI for OS were estimated based on the data extracted from the K-M curves or survival rate.

Quality assessment was performed by the modified Newcastle-Ottawa Scale on all 18 studies, as displayed in Table 2. Most studies were ranked five stars, suggesting acceptable overall quality of the selected studies. Of note, in the association of high MACC1 expression with survival, no study attempted to control other confounding prognostic factors, such as variation of treatment.

### 3.3. Primary Analysis for Prognostic Effect of High versus Low MACC1 in Digestive System Neoplasms

**Figure 2:**
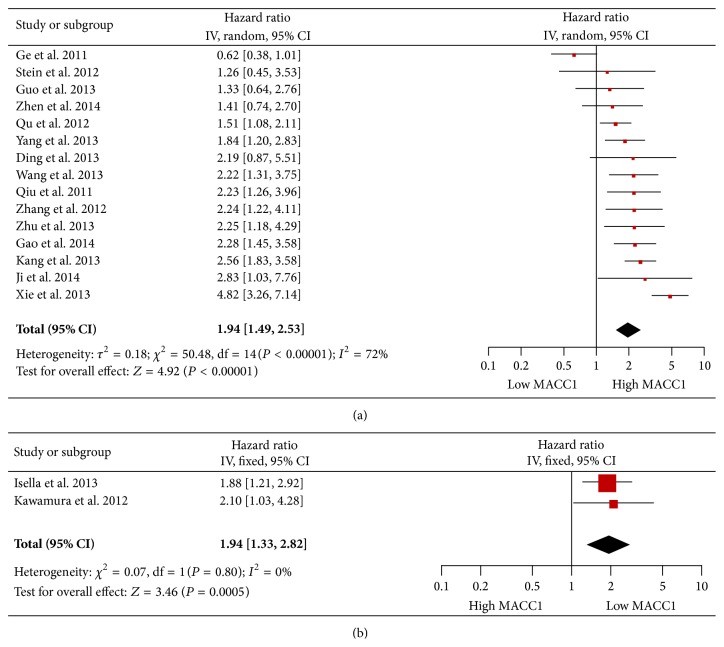
Forest plot and meta-analysis of studies evaluating hazard ratios (HRs) for clinical outcomes of high MACC1 expression versus low expression in digestive system neoplasms. (a) Pooled HR and 95% CI for overall survival (OS). (b) Pooled HR and 95% CI for relapse-free survival (RFS). A fixed-effect or random-effect model was used for data pooling in accordance with heterogeneity. Heterogeneity was accessed by *χ* ^2^ test and demonstrated by *I* ^2^ and *p* value (*I* ^2^ < 25%: no heterogeneity; *I* ^2^ = 25–50%: moderate heterogeneity; *I* ^2^ > 50% or *p* > 0.01: large or extreme heterogeneity).

At first, meta-analysis for the correlation between MACC1 expression and the primary outcome OS in 15 of all included studies was performed. Considering the rather large heterogeneity (*p* < 0.00001, *I* ^2^ = 72%), the pooled HR was calculated by a random-effect model. The result suggests that patients with high MACC1 expression tend to have a significantly poorer OS (HR = 1.94, 95% CI: 1.49–2.52) compared to those with low MACC1 expression (Figure 2(a)).

Additionally, MFS, RFS, and DFS were considered as secondary outcomes. The HRs and corresponding 95% CI for each aforementioned clinical outcome are also demonstrated in Table 1. Consistent with the pooled OS, the result of pooled RFS also indicated that high MACC1 expression is significantly associated with worse clinical outcome (HR = 1.94, 95% CI: 1.33–2.82) and *I* ^2^ = 0% suggests that there is no heterogeneity (Figure 2(b)). In terms of MFS and DFS, we failed to calculate the pooled HR due to inadequate number of studies.

### 3.4. Subgroup Analyses in Multiple Settings for MACC1 Expression and OS in Digestive System Neoplasms

Results of subgroup analyses for pooled OS were demonstrated in Table 3. Firstly, we categorized the included studies based on different methods in measuring MACC1 expression. In the qRT-PCR subgroup, a significantly poorer OS (HR = 1.80, 95% CI: 1.46–2.22) was observed in patients with high MACC1 expression and there was no heterogeneity detected (*p* = 0.53, *I* ^2^ = 0%). Despite the significant heterogeneity (*p* < 0.01, *I* ^2^ = 80%), the same conclusion can be drawn from the IHC subgroup (HR = 2.00, 95% CI: 1.33–2.99). Secondly, subgroup analyses for anatomic structure were performed. As expected, the subgroup analysis for cancers in gastrointestinal tract also consistently (*p* < 0.01, *I* ^2^ = 72%) identified high MACC1 expression as a risk factor for poor OS (HR = 1.62, 95% CI: 1.10–2.40). Moreover, the result in the subgroup of cancers in nongastrointestinal tract was statistically significant (HR = 2.29, 95% CI: 1.91–2.74) with no heterogeneity (*p* < 0.01, *I* ^2^ = 71%) as well. In addition, we conducted subgroup analysis for different cancer subtypes providing that at least two studies were available for each subgroup. Statistical significance was seen in all subgroups in this setting, including CRC subgroup (HR = 2.17, 95% CI: 1.68–2.81), GC subgroup (HR = 1.21, 95% CI: 0.54–2.75), and HCC subgroup (HR = 2.29, 95% CI: 1.91–2.74). Heterogeneity was detected in this setting as well (Table 3).

### 3.5. Sensitivity Analysis

Sensitivity analysis was carried out to evaluate the stability of all the pooled datasets. No study was found to remarkably affect either the pooled HRs for OS or the pooled HR for RFS. Surprisingly, two studies contributed the most to the observed heterogeneity. One is the study on GC by Ge et al., 2011 [17], and the other is the study on HCC by Xie et al., 2013 [22]. After excluding these two studies and recalculating the pooled HRs, no heterogeneity was detected.

### 3.6. Metaregression

In the end, since there was extreme heterogeneity among the 15 studies selected for pooled OS, a metaregression analysis was performed to recognize the source of heterogeneity by publication year, cancer subtypes, and MACC1 measurement in the OS dataset (Table S3 in Supporting Information). The results indicated that publication year and cancer subtypes had no contribution to the observed heterogeneity (*p* = 0.207; *p* = 0.466, resp.). However, the methods of MACC1 measurement contribute significantly to the heterogeneity (*p* = 0.019).

### 3.7. Publication Bias Assessment

**Figure 3:**
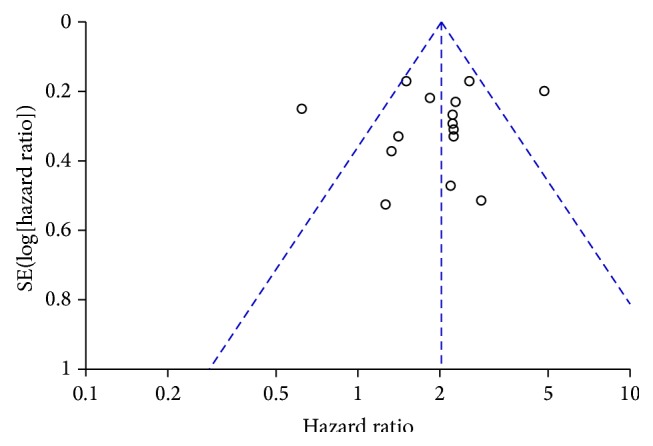
Funnel plot for publication bias assessment of pooled overall survival (OS) in this meta-analysis. The existence of publication bias is determined by the degree of the figure's symmetry.

Visual assessment of a funnel plot provided no evidence of publication bias for all 15 included studies for pooled OS (Figure 3). Furthermore, Egger's test was conducted for more precise publication bias assessment and the result indicated no publication bias for pooled OS as well (*p* = 0.788). Considering that less than 10 eligible studies were included for pooled MFS, RFS, and DFS, tests for funnel plot asymmetry and Egger's test were not applied for publication bias assessment in terms of these clinical outcomes [31].

## 4. Discussion

Because of the high morbidity and mortality of digestive system neoplasms, researchers have been dedicated to identify available new prognostic markers to achieve better clinical decision-making regarding therapy and outcomes in decades. To evaluate the prognostic significance of a potential biomarker, it is of great necessity to gather and synthesize as much information as possible on this topic to acquire a relatively large sample size and to conduct comprehensive evaluation [2]. As a novel oncogene, MACC1 overexpression has been associated with poor clinical outcome of patients with digestive system neoplasms [3, 5, 9, 10]. A recent meta-analysis that included a total of 20 eligible studies with patients also showed that overexpression of MACC1 was significantly associated with poorer survival in solid tumors [11], whereas subgroup analysis was not performed in this meta-analysis to validate the correlation between MACC1 expression and survival in patients with digestive system neoplasms. Whether high MACC1 expression is correlated with poorer clinical outcomes needs further proof and the prognostic value of MACC1 in digestive system neoplasms has not been validated by systematic review and meta-analysis. Therefore, we examined the correlation between high levels of MACC1 and OS in patients with digestive system neoplasm extracted from 18 eligible studies via systematic review and meta-analysis. The results suggested that high MACC1 expression is significantly correlated with both poorer OS and poorer RFS in patients with digestive system neoplasms. In terms of MFS and DFS, Stein et al. in 2009 [3] first reported that high MACC1 expression indicates poorer MFS in patients with CRC and Isella et al. in 2013 [25] reported that high MACC1 expression is significantly correlated with poorer DFS in patients with CRC. So far, there are no more studies reporting the correlation between MACC1 expression and MFS or DFS. Consequently, we failed to calculate the pooled HRs for these two clinical outcomes. Yet the results of both studies support the systematic evidence yielded from this meta-analysis which is that high MACC1 expression leads to poorer clinical outcomes in patients with digestive system neoplasms. Furthermore, both subgroup analysis and metaregression analyses were carried out to clarify the potential heterogeneity. Both the pooled HRs after eliminating the suspected source of heterogeneity and HRs of subgroup analyses verified the correlation between high MACC1 expression and poorer OS, which strengthens the evidence of this meta-analysis.

In our initial analysis, significant heterogeneity was detected by *χ* ^2^ test (*p* < 10^−5^; *I* ^2^ = 72%). Consequently, subgroup analyses were performed to further explore the source of this observed heterogeneity. As shown in Table 3, the subgroup analyses for detection method, anatomic structure, and cancer subtype demonstrated that the pooled HRs of all subgroups except GC group were statistically significant. We found that one of the studies in GC group shows that high MACC1 expression indicates better prognosis of GC patients [17], which is the opposite from the other two studies. Considering the notable inconsistency, we noticed that the sample size in this study was only 128 patients and the positive staining of MACC1 was defined as >20% cells having positive immunoreactivity, which, in our opinion, is less subjective than the immunohistochemistry of MACC1 with both the staining intensity score and the rate of positive cells. Therefore, we were convinced that the result from this study was susceptible and more studies were required for determining the prognostic value of MACC1 in GC.

Furthermore, significant heterogeneity was also detected in the subgroup of IHC, gastrointestinal tract, nongastrointestinal tract, GC, and HCC. Therefore, we conducted metaregression to test the heterogeneity. The results excluded publication year and cancer subgroup as contributing factor to the observed heterogeneity. However, the methods of MACC1 measurement appeared to be a potential source for heterogeneity (Table S3 in Supporting Information). Currently, there are multiple methods to measure MACC1 expression and the optimal cut-off point to assign MACC1 expression at present. Even though the pooled HRs of both qRT-PCR group and IHC group were statistically significant and both consistently identified high MACC1 expression as a contributor to poor survival, the diversity of cut-off point determination was still quite distinguishable. Meanwhile, we identified two studies contributing the most to the heterogeneity in the sensitivity analysis, both of which measured MACC1 expression via IHC [17, 22]. Since qRT-PCR and IHC measure MACC1 expression on different molecular level and the difference of tissue processing and result interpretation, the potential bias and confounding factors still exist. Taking the results of metaregression analysis together, side by side comparison between different methods of measuring MACC1 expression seems necessary.

Moreover, it is worth noticing that no studies on PC were included in this meta-analysis, which made liver the only digestive gland for the subgroup analysis. Even though the pooled HRs for cancer subtype were consistent with the results from other subgroup analyses, this limitation should not be ignored. As we know, pancreatic cancer has been considered as one of the most refractory cancers in digestive system. Since we verified the positive correlation between high MACC1 expression and adverse clinical outcome in this meta-analysis, a well-designed trial with adequate sample size should be conducted for analysis of the impact of MACC1 expression on the survival of PC patients in the future.

The initial assessment by the funnel plot indicated no publication bias, which was further confirmed by Egger's test. However, the result should still be interpreted with caution in that this meta-analysis might not be completely bias-free. As is known, publication bias can still be a major problem on all meta-analyses, including those evaluating the prognostic value of a biomarker since positive results tend to be easier to get published. Thus it is very important to be aware of this problem when evaluating the reliability of a positive report on the prognostic value of MACC1.

In addition, we noticed that the retrospective design was adopted by most studies in this meta-analysis. Compared to randomized controlled trials (RCTs), the observational cohort of most included studies would to some extent increase the risk of potential confounding factors. For example, gender, TNM stage, histology type, races, and age were not well controlled in most included studies which might cause heterogeneity. Besides, it should be noticed that 14 of the 15 included studies were performed in the Asian population, which indicates the geographic limitation in this meta-analysis even though the inclusion criteria were strictly applied.

As a whole, our meta-analysis provides relatively comprehensive evidence on the role of MACC1 expression level in prognostic value in patients with digestive system neoplasms. On one hand, this is the first meta-analysis to integrate studies analyzing the association of MACC1 expression and clinical outcome in digestive system neoplasms; on the other hand, the subgroup analyses in multiple perspectives enhance the reliability and strengthen the evidence of this meta-analysis. To conclude, the evidence provided by this systematic review and meta-analysis suggests that MACC1 might be served as a prognostic biomarker for digestive system neoplasms. Yet large multicenter random control trials should be conducted in the future as to verify the prognostic value of MACC1 in digestive system neoplasms.

## Supplementary Material

Table S1 PRISMA 2009 Checklist. The page reference numbers for each item required in this checklist are provided for transparent reporting of this meta-analysis.
Table S2 Newcastle–Ottawa quality assessment scale. The Newcastle-Ottawa Quality Assessment Scale (NOS) for non-RCT study is used for quality assessment in this meta-analysis. The scale includes eight items which allows for assessment of patient selection, study comparability, and outcome of interest. A study with five or more stars was considered as ahigh-quality study.
Table S3 Results of Meta Regression. Meta-regression analysis was performed to recognize the source of heterogeneity by publication year, cancer subtypes and MACC1 measurement in the OS dataset and a *p* < 0.05 indicates strong evidence for a certain factor contributing to the observed heterogeneity.

## Figures and Tables

**Table 1:** Summary of eligible studies included for meta-analysis.

	Study design	Number of patients	Cancer subtype	TNM staging	Sample	MACC1 detection	MACC1 cut-off	High MACC1 proportion	Outcome	HR obtainment	HR	95% CI
Stein et al., 2012 [12, 13]	C	294	CRC	I–IV	Blood	qRT-PCR	Median value	147/294	OS	Estimated	1.260	0.450–3.530
Zhang et al., 2012 [14]	R	90	CRC	I–IV	Tissue	IHC	Score ≥ 5	78/90	OS	Estimated	2.240	1.220–4.100
Kang et al., 2013 [15]	R	317	CRC	I–IV	Tissue	IHC	Positive cells > 20%	176/317	OS	Reported	2.560	1.830–3.570
Zhen et al., 2014 [16]	R	323	CRC	I–IV	Tissue	IHC	Staining score = 6	169/323	OS	Reported	1.410	0.737–2.699
Ge et al., 2011 [17]	R	128	GC	I–IV	Tissue	IHC	Positive cells ≥ 20%	61/128	OS	Reported	0.621	0.380–1.014
Guo et al., 2013 [18]	R	98	GC	I–IV	Tissue	IHC	Score > 3	60/98	OS	Estimated	1.330	0.640–2.760
Wang et al., 2013 [5]	R	361	GC	I–IV	Tissue	IHC	Score ≥ 3	280/361	OS	Reported	2.217	1.311–3.049
Zhu et al., 2013 [9]	R	85	EC	I–III	Tissue	IHC	Score > 2	47/85	OS	Reported	2.250	1.180–4.280
Yang et al., 2013 [19]	R	120	HCC	N/A	Tissue	qRT-PCR	Median value	60/120	OS	Reported	1.842	1.201–2.514
Qiu et al., 2011 [10]	R	128	HCC	I–III	Tissue	qRT-PCR	Threshold value	57/128	OS	Reported	2.230	1.257–3.957
Qu et al., 2012 [20]	R	234	HCC	N/A	Tissue	qRT-PCR	Value > 0.006 73	105/234	OS	Reported	1.508	1.079–1.835
Ding et al., 2013 [21]	R	66	HCC	I–IV	Tissue	IHC	Positive cells > 50%	33/66	OS	Estimated	2.190	0.870–5.530
Xie et al., 2013 [22]	R	308	HCC	I–IV	Tissue	IHC	Score > 6	126/308	OS	Reported	4.823	3.257–6.893
Ji et al., 2014 [23]	R	60	HCC	I–IV	Tissue	IHC	Score ≥ 3	41/60	OS	Reported	2.827	1.030–7.759
Gao et al., 2014 [24]	R	160	HCC	N/A	Tissue	qRT-PCR	ROC curve	72/160	OS	Reported	2.280	1.453–3.580
Stein et al., 2009 [3]	C	60	CRC	I–III	Tissue	qRT-PCR	Median value	30/60	MFS	Estimated	3.340	1.820–6.130
Isella et al., 2013 [25]	R	64	CRC	N/A	Tissue	qRT-PCR	>−1.30	51/64	RFS DFS	Estimated Reported	1.880 7.274	1.210–2.920 1.658–31.905
Kawamura et al., 2012 [26]	R	52	CRC	II-III	Tissue	qRT-PCR	0.261	18/52	RFS	Estimated	2.100	1.030–4.300

Study design is described as consecutive patients (C) or retrospective (R). Cancer subgroups include colorectal cancer (CRC), gastric cancer (GC), esophageal cancer (EC), and hepatocellular cancer (HCC). Clinical outcomes include overall survival (OS), metastasis-free survival (MFS), relapse-free survival (RFS), and disease-free survival (DFS). MACC1 measurement is categorized as quantitative real-time polymerase chain reaction (qRT-PCR) and immunohistochemistry (IHC) group.

**Table 2:** Summary of Newcastle-Ottawa quality assessment scale.

	Newcastle-Ottawa scale category			Total
	Selection	Comparability	Outcome	
Stein et al., 2012 [12, 13]	⋆⋆⋆	/	⋆⋆	⋆⋆⋆⋆⋆
Zhang et al., 2012 [14]	⋆⋆	/	⋆⋆	⋆⋆⋆⋆
Kang et al., 2013 [15]	⋆⋆	/	⋆⋆	⋆⋆⋆⋆
Zhen et al., 2014 [16]	⋆⋆⋆	/	⋆⋆	⋆⋆⋆⋆⋆
Stein et al., 2009 [3]	⋆⋆⋆	/	⋆⋆	⋆⋆⋆⋆⋆
Isella et al., 2013 [25]	⋆⋆	/	⋆⋆	⋆⋆⋆⋆
Kawamura et al., 2012 [26]	⋆⋆	/	⋆⋆	⋆⋆⋆⋆
Ge et al., 2011 [17]	⋆⋆⋆	/	⋆⋆	⋆⋆⋆⋆⋆
Guo et al., 2013 [18]	⋆⋆⋆	/	⋆⋆	⋆⋆⋆⋆⋆
Wang et al., 2013 [5]	⋆⋆⋆	/	⋆⋆	⋆⋆⋆⋆⋆
Zhu et al., 2013 [9]	⋆⋆⋆	/	⋆⋆	⋆⋆⋆⋆⋆
Yang et al., 2013 [19]	⋆⋆⋆	/	⋆⋆	⋆⋆⋆⋆⋆
Qiu et al., 2011 [10]	⋆⋆⋆	/	⋆⋆	⋆⋆⋆⋆⋆
Qu et al., 2012 [20]	⋆⋆⋆	/	⋆⋆	⋆⋆⋆⋆⋆
Ding et al., 2013 [21]	⋆⋆	/	⋆⋆	⋆⋆⋆⋆
Xie et al., 2013 [22]	⋆⋆⋆	/	⋆⋆	⋆⋆⋆⋆⋆
Ji et al., 2014 [23]	⋆⋆⋆	/	⋆⋆	⋆⋆⋆⋆⋆
Gao et al., 2014 [24]	⋆⋆⋆	/	⋆⋆	⋆⋆⋆⋆⋆

The scale includes eight items in total with four items in selection category, one item in comparability category, and three items in outcome category. Stars were given to high-quality elements. Having five or more stars is considered good quality.

**Table 3:** Summary of subgroup analyses in multiple settings for pooled OS.

Subgroup	Number of studies	Pooled hazard ratio	95% CI	Heterogeneity (*p* value; *I* ^2^)	*p* value
Detection method					
IHC	10	2.00	[1.33, 2.99]	<0.01; 80%	<0.05
qRT-PCR	5	1.80	[1.46, 2.22]	0.53; 0	<0.05
Anatomic structure					
Gastrointestinal tract	8	1.62	[1.10, 2.40]	<0.01; 72%	<0.05
Nongastrointestinal tract	7	2.29	[1.91, 2.74]	<0.01; 71%	<0.05
Cancer subtype					
CRC	4	2.08	[1.52, 2.85]	0.29; 19%	<0.05
GC	3	1.21	[0.54, 2.75]	<0.01; 84%	0.64
HCC	7	2.29	[1.91, 2.74]	<0.01; 71%	<0.05

Subgroup analyses were performed when there were at least two studies in certain subgroup.
