# Supplementary material for: Prognostic Value of MACC1 in Digestive System Neoplasms: A Systematic Review and Meta-Analysis
Source: Biomed Res Int. 2015 May 19;2015:252043. doi: 10.1155/2015/252043 (PMC4452247; doi:10.1155/2015/252043)
Supplement: Supplementary file 1 — Table S1 PRISMA 2009 Checklist. The page reference numbers for each item required in this checklist are provided for transparent reporting of this meta-analysis. Table S2 Newcastle–Ottawa quality assessment scale. The Newcastle-Ottawa Quality Assessment Scale (NOS) for non-RCT study is used for quality assessment in this meta-analysis. The scale includes eight items which allows for assessment of patient selection, study comparability, and outcome of interest. A study with five or more stars was considered as ahigh-quality study. Table S3 Results of Meta Regression. Meta-regression analysis was performed to recognize the source of heterogeneity by publication year, cancer subtypes and MACC1 measurement in the OS dataset and a p < 0.05 indicates strong evidence for a certain factor contributing to the observed heterogeneity. [file 252043.f1.pdf]

PRISMA 2009 Checklist

Section/topic	#	Checklist item	Reported on page #
**TITLE**			
Title	1	Identify the report as a systematic review, meta-analysis, or both.	1
**ABSTRACT**			
Structured summary	2	Provide a structured summary including, as applicable: background; objectives; data sources; study eligibility criteria, participants, and interventions; study appraisal and synthesis methods; results; limitations; conclusions and implications of key findings; systematic review registration number.	2
**INTRODUCTION**			
Rationale	3	Describe the rationale for the review in the context of what is already known.	3-4
Objectives	4	Provide an explicit statement of questions being addressed with reference to participants, interventions, comparisons, outcomes, and study design (PICOS).	3-4
**METHODS**			
Protocol and registration	5	Indicate if a review protocol exists, if and where it can be accessed (e.g., Web address), and, if available, provide registration information including registration number.	N/A
Eligibility criteria	6	Specify study characteristics (e.g., PICOS, length of follow-up) and report characteristics (e.g., years considered, language, publication status) used as criteria for eligibility, giving rationale.	4-5
Information sources	7	Describe all information sources (e.g., databases with dates of coverage, contact with study authors to identify additional studies) in the search and date last searched.	4
Search	8	Present full electronic search strategy for at least one database, including any limits used, such that it could be repeated.	4
Study selection	9	State the process for selecting studies (i.e., screening, eligibility, included in systematic review, and, if applicable, included in the meta-analysis).	4-5
Data collection process	10	Describe method of data extraction from reports (e.g., piloted forms, independently, in duplicate) and any processes for obtaining and confirming data from investigators.	5
Data items	11	List and define all variables for which data were sought (e.g., PICOS, funding sources) and any assumptions and simplifications made.	5
Risk of bias in individual studies	12	Describe methods used for assessing risk of bias of individual studies (including specification of whether this was done at the study or outcome level), and how this information is to be used in any data synthesis.	5
Summary measures	13	State the principal summary measures (e.g., risk ratio, difference in means).	6
Synthesis of results	14	Describe the methods of handling data and combining results of studies, if done, including measures of consistency (e.g., I^2) for each meta-analysis.	6

PRISMA 2009 Checklist

Section/topic	#	Checklist item	Reported on page #
Risk of bias across studies	15	Specify any assessment of risk of bias that may affect the cumulative evidence (e.g., publication bias, selective reporting within studies).	**6-7**
Additional analyses	16	Describe methods of additional analyses (e.g., sensitivity or subgroup analyses, meta-regression), if done, indicating which were pre-specified.	**6-7**
**RESULTS**			
Study selection	17	Give numbers of studies screened, assessed for eligibility, and included in the review, with reasons for exclusions at each stage, ideally with a flow diagram.	**7**
Study characteristics	18	For each study, present characteristics for which data were extracted (e.g., study size, PICOS, follow-up period) and provide the citations.	**7-9**
Risk of bias within studies	19	Present data on risk of bias of each study and, if available, any outcome level assessment (see item 12).	**9**
Results of individual studies	20	For all outcomes considered (benefits or harms), present, for each study: (a) simple summary data for each intervention group (b) effect estimates and confidence intervals, ideally with a forest plot.	**Figure 2.**
Synthesis of results	21	Present results of each meta-analysis done, including confidence intervals and measures of consistency.	**9-10**
Risk of bias across studies	22	Present results of any assessment of risk of bias across studies (see Item 15).	**10**
Additional analysis	23	Give results of additional analyses, if done (e.g., sensitivity or subgroup analyses, meta-regression [see Item 16]).	**10-11**
**DISCUSSION**			
Summary of evidence	24	Summarize the main findings including the strength of evidence for each main outcome; consider their relevance to key groups (e.g., healthcare providers, users, and policy makers).	**11-12**
Limitations	25	Discuss limitations at study and outcome level (e.g., risk of bias), and at review-level (e.g., incomplete retrieval of identified research, reporting bias).	**12-14**
Conclusions	26	Provide a general interpretation of the results in the context of other evidence, and implications for future research.	**14**
**FUNDING**			
Funding	27	Describe sources of funding for the systematic review and other support (e.g., supply of data); role of funders for the systematic review.	**online submission system**

From: Moher D, Liberati A, Tetzlaff J, Altman DG, The PRISMA Group (2009). Preferred Reporting Items for Systematic Reviews and Meta-Analyses: The PRISMA Statement. PLoS Med 6(6): e1000097. doi:10.1371/journal.pmed1000097

For more information, visit: www.prisma-statement.org.

Table S1. Newcastle–Ottawa quality assessment scale

Selection

1) Representativeness of the exposed cohort

a) truly representative of the average 'patients with digestive system neoplasm' in the community (★)

b) somewhat representative of the average 'patients with digestive system neoplasm' in the community (★)

c) selected group of users (eg. nurses, volunteers)

d) no description of the derivation of the cohort

2) Selection of the non exposed cohort

a) drawn from the same community as the exposed cohort (★)

b) drawn from a different source

c) no description of the derivation of the non exposed cohort

3) Ascertainment of exposure (Proof of digestive system neoplasm and MACC1 evaluation)

a) secure record (eg. surgical records) (★)

b) structured interview (★)

c) written self report

d) no description

4) Demonstration that outcome of interest was not present at start of study

a) yes (★)

b) no

Comparability

1) Comparability of cohorts on the basis of the design or analysis

a) study controls for ‘tumor stage or treatment’ (★)

b) study controls for any additional factor (Age, gender, TNM stage, treatment etc) (★)

Outcome

1) Assessment of outcome (Death)

a) independent blind assessment (★)

b) record linkage (★)

c) self report

d) no description

2) Was follow-up long enough for outcomes to occur

a) yes (★)

b) no

3) Adequacy of follow up of cohorts

a) complete follow up (all subjects accounted for) (★)

b) subjects lost to follow up unlikely to introduce bias (small number lost)- >75% follow up; or description provided of those lost) (★)

c) follow up rate < 75% and no description of those lost

d) no statement

Note: A study can be awarded a maximum of one star for each numbered item within the Selection and Outcome categories. A maximum of two stars can be given for Comparability.
